# Food Value of Mealworm Grown on *Acrocomia aculeata* Pulp Flour

**DOI:** 10.1371/journal.pone.0151275

**Published:** 2016-03-14

**Authors:** Ariana Vieira Alves, Eliana Janet Sanjinez-Argandoña, Adelita Maria Linzmeier, Claudia Andrea Lima Cardoso, Maria Lígia Rodrigues Macedo

**Affiliations:** 1 Faculty of Exact Sciences and Technology, Federal University of Grande Dourados, Dourados, Mato Grosso do Sul, Brazil; 2 Federal University of Fronteira Sul, Realeza, Paraná, Brazil; 3 Course of Chemistry, State University of Mato Grosso do Sul, Dourados, Mato Grosso do Sul, Brazil; 4 Laboratory of Protein Purification and Biological Functions, Departament of Natural Science, Federal University of Mato Grosso do Sul, Campo Grande, Mato Grosso do Sul, Brazil; CNRS, FRANCE

## Abstract

Insects have played an important role as human food throughout history, especially in Africa, Asia and Latin America. A good example of edible insects is the mealworm, *Tenebrio molitor* Linnaeus, 1758 (Coleoptera, Tenebrionidae), which are eaten in Africa, Asia, the Americas and Australia. This species is easily bred in captivity, requiring simple management. The bocaiuva (*Acrocomia aculeata* (Jacq.) Lodd) is an abundant palm tree found in the Brazilian Cerrado, providing fruits with high nutritional value. The aim of this work was to determine the chemical composition of *T*. *molitor* grown in different artificial diets with bocaiuva pulp flour. The nutritional composition, fatty acid composition, antioxidant activity, trypsin activity and anti-nutritional factors of larvae were analyzed. The results showed that mealworms grown on artificial diet with bocaiuva are a good source of protein (44.83%) and lipid (40.45%), with significant levels of unsaturated fatty acids (65.99%), antioxidant activity (4.5 μM Trolox/g of oil extracted from larvae) and absence of anti-nutritional factors. This study indicates a new source of biomass for growing mealworms and shows that it is possible to breed mealworms in artificial diet with bocaiuva flour without compromising the nutritional quality of the larvae.

## Introduction

Insects have played an important role in human food throughout history, especially in Africa, Asia and Latin America [1,2]. More than 2.000 species of edible insects have been cataloged around the world [3], including 135 in Brazil [4]. A rapid increase in the human population is expected in the second half of the XXI century, which will lead to lower availability of food, especially animal protein [5,6].

According to the Food and Agriculture Organization of the United Nations (FAO) [7], in 2050 we will be nine billion people, which will require more food sources. In 2013, after the International Conference on Forests for Food Security and Nutrition, FAO published a report [8] encouraging insect consumption as a way to fight hunger and promote food security; insects are a source of good nutritional quality protein for humans.

The introduction of new food items in the human diet, despite challenges, has precedent, i.e., negative impressions on certain types of foods can be reconsidered. Consumers found that certain cheeses with strong flavor and smell can be tasteful, and the consumption of live animals (e.g. oysters) and raw meat (e.g. sashimi, carpaccio) is now common [9].

It seems quite illogical that eating invertebrates such as lobster and shrimp (that feed on decomposing material) is considered normal for human consumption; while the consumption of insects (also invertebrates and arthropods, some exclusively herbivores) is seen with prejudice [10]. The information that insects have high nutritional value and can be raised in a sustainable manner, may break down prejudice barriers and allow the use of insects as a food source or food supplement.

Insects are very efficient in organic matter biotransformation (high feed conversion ratio) becoming a high nutritional value biomass [11,12]. For example, insect larvae (various species) bred in captivity, in pre-established conditions, turn plant biomass into animal biomass up to 10 times more efficiently than cattle [8], mainly due to their poikilothermic characteristic ("cold-blooded animals"). They use less energy to maintain body heat, because they use the environment to regulate body temperature [12].

Thus, insect breeding can provide sustainable food production with lower environmental impact than the conventional livestock [13]. A quarter of the world's land is used today for the breading 1.7 billion cattle, while a third of arable land is used to plant grains that sustain livestock [14]. Mass production of insects for human consumption using industrial methods is technically feasible. To meet the protein needs of 100 people breeding beetles, would require only 40m^3^ [15]. Recent examples of edible insects being bred commercially for human consumption include the domestic cricket (*Acheta domesticus* Linnaeus, 1758), the palm weevil (*Rhynchophorus ferrugineus* Olivier, 1790) and the water cockroach (*Lethocerus indicus* Lepeletier & Serville, 1825—Belostomatidae) in Thailand and water beetles in China [15].

Among edible insect species stands out the mealworms *Tenebrio molitor* Linnaeus, 1758 (Coleoptera, Tenebrionidae), since it is currently consumed by humans, especially in Africa, Asia, the Americas and Australia. This is an insect species that has one of the highest amounts of protein (from 47.76 to 53.13%) and lipids (27.25 to 38.26%), with energy contributions varying from 379 to 573 kcal/100g [16]. Considering a daily energy value for an adult of 2000 kcal/day, 100 g of *T*. *molitor* meet approximately a quarter of the daily energy needed [17,18,19]. Therefore, the energy intake from insects may be important for food security.

*T*. *molitor* is among the largest beetles that infest food products in warehouses, mainly grain warehouses. This species begin to lay eggs from 4 to 17 days after copulation. A single female can generate an average of 500 eggs. The embryonic development lasts from 4 to 6 days, which can be accelerated with a slight increase in temperature (25 to 27°C). Larval period is about 3 months; at this stage, the insect is consumed. An average mature larva weighs 0.2 g and measures 25-35mm long. After this phase, the larva turns into a pupa, a stage that lasts 5 to 6 days and culminates in an adult individual [20].

Breeding insects in captivity requires the administration of artificial diets usually consisting of leaves and grains. In the Brazilian Cerrado, the palm *Acrocomia aculeata* (Jacq.) Lodd. ex Mart. (Arecaceae), known as bocaiuva or macaúba is abundant and provides fruit with high nutritional value [21,22,23] and anti-inflammatory properties [24,25], mainly due to its carotenoids and fatty acids.

The bocaiuva pulp oil is composed predominantly monounsaturated of fatty acids, especially oleic acid. The unsaturated fatty acids play important roles in the human body, for example, the maintenance of the immune system against inflammatory processes [25].

Alternative diets for *T*. *molitor* were prepared with bocaiuva flour aiming to increase the concentration of their unsaturated fatty acids. Comparing the nutritional value of the larvae fed different diets can indicate new biomass sources to increase the mealworm nutritional value. In this context, the aim of this work was to determine the chemical composition of *T*. *molitor* larvae grown on different diets with bocaiuva pulp flour.

## Material and Methods

### Material

Mealworms were purchased from a private breeder (Atraki, São Paulo-SP, Brazil). The second generation of larvae were grown in four flour diets consisting of: wheat, soybean, bocaiuva pulp and dehydrated bocaiuva kernels. Wheat and soybean flours were acquired in the street market of Dourados-MS, Brazil. The bocaiuva fruits were collected in Dourados, MS; they were cleaned, pulp and kernel were separated, they were separately dried in an oven with air circulation separately at 45°C for 48 hours. Thereafter they were separately crushed and the pulp was sieved in tamis with mesh aperture of 355μm, obtaining the corresponding flour.

### Mealworm nutrition

The acquired mealworms were kept in polystyrene boxes (40x30x25cm) for 30 days to complete their life cycle. The feed for the second generation of mealworms were divided into four diets: (A) 50% wheat flour, 50% soybean flour (control diet); (B) 50% control diet and 50% bocaiuva pulp flour; (C) 50% Control diet and 50% ground bocaiuva kernel; and (D) 50% bocaiuva pulp flour and 50% ground bocaiuva kernel.

Approximately 400 mealworms were placed per box, according to the food managing of each diet (A, B, C and D). Average temperature was 25°C, relative humidity of 80% and photoperiod of 10h light (0,18 Klux) and 14h dark (0 Klux). After 90 days, the larvae were collected and frozen at -6°C, and stored at this temperature until analysis time.

### Nutritional composition

The nutritional composition of the four diets (A, B, C and D), and the mealworm fed diets A and B were determined. Evaluations were performed on moisture contents in an oven [25]; fixed mineral residue (ashes) in an oven at 550°C [25]; lipids extraction with petroleum ether using Soxhlet equipment [25]; protein content, determining the nitrogen present in the samples by the Kejldahl method [25] using a conversion factor of 6.25; and fibers by acid and alkaline extraction [26]. The carbohydrates evaluation was performed by difference (100g sample–moisture–ashes–lipid–protein–fibers). The energy value was calculated using the *Atwater coefficient*, using 4 kcal/g of sample for proteins and carbohydrates and 9 kcal/g for lipids [27].

### Fatty acid composition

The oil from mealworms fed diets A and B was extracted according to the Bligh & Dyer method [28]. The transesterification of triglycerides were performed with approximately 50 mg of extracted lipid matters transferred to 15 ml falcon tubes, to which were added 2 ml of n-heptane. The mixture was stirred until complete dissolution of the fatty matter and 2 ml of KOH and 2 mol/l methanol were then added. The mixture was stirred for about 5 minutes; after phases separated, 1 ml of the upper phase (heptane and methyl esters of fatty acids) was transferred to 1.5 ml Eppendorf vials. The vials were hermetically closed, protected from light and stored in a freezer at -18°C for further chromatographic analysis.

The fatty acid composition was determined by gas chromatography using a gas chromatograph with flame ionization detector (GC-FID). A capillary column of 100m x 0.25 mm x fused silica 0.20 μm (SP-2560) was used for elution. The oven temperature was programmed to begin at 100°C for 1 min, then raised to 170°C at 6.5°C/min.

Afterwards, another increase from 170 to 215°C was performed at 2.75°C/min, and maintained for 12 min. A last elevation was performed from 215°C to 230°C at 40°C/min. The injector and detector temperatures were 270°C and 280°C, respectively.

Samples (0.5 μL) were injected in "split" (1:20), using nitrogen as a carrier gas at a drag rate of 1 ml/min. The identification of methyl esters of fatty acids was performed by comparison with the retention times of the sample compounds with the standards (Sigma) eluted under the same conditions of the samples.

### Antioxidant activity analysis

An extract was prepared from a mixture of 1 g of previously extracted mealworm oil and 50 ml of hydromethanol solution (50%). After resting for 60 min, the material was centrifuged (4000 rpm) for 15 min and the supernatant removed. Acetone (40 ml at 70%) was added to the pellet to perform the second extraction, following the first extraction procedure. The supernatants from both extractions were mixed, transferred to a flask and added distilled water to complete the volume of 100 ml, obtaining the extract.

The radical ABTS^•+^ (2, 2-Azino BIS-3-ethylbenzo thiazoline 6 sulfonic acid diammoninum) was formed by the reaction of ABTS^•+^ (7 mM) with potassium persulfate (140 mM), the mixture reacted at room temperature for 16 h with absence of light, obtaining the radical solution. The radical solution was diluted in ethanol until absorbance of 0.70 (± 0.05) at 734 nm (spectrophotometer Biospectro) for upcoming analyses. Samples (30 μl) were added to 3 ml of the ABTS^•+^ diluted solution and the mixture absorbance was registered after 6 minutes. The antioxidant activity was calculated using the standard curve of 6-hydroxy-2,5,7,8-tetrametilchroman-2-carboxylic acid (Trolox). The standard curve was prepared from Trolox ethanolic solutions at concentrations 100; 500; 1000; 1500 and 2000 μM [29]. The results were expressed as mM of Trolox/g of extract. Each determination was performed in triplicate.

### Analysis of tryptic and chymotryptic activities

The tryptic and chymotryptic analyses were carried out in microplates [30]. The assay utilizes the hydrolysis of chromogenic substrates BApNA (N α-Benzoyl-DL-Arginine p-nitroanilide) to trypsin and SAAPFPNA (Succynil Alanine PF p-nitroanilide) for chymotrypsin.

The tryptic activity of mealworms fed diet A (50% wheat flour and 50% soybean flour) was assessed by incubating the samples with Tris-HCl 50 mM, pH 8.0 to a final volume of 70 μl. After the substrate addition, the test time was 30 min at 37°C. Results were expressed as nmol/BApNA/min and IU/ml. The chymotryptic activity of larvae was assessed by incubating the samples with Tris-HCl 50 mM, pH 8.0 to a final volume of 100 μl. After the substrate addition, the test time was 10 minutes at 37°C and the reaction read in the Multiskan Go Microplate Reader at 410 nm. This analysis results were expressed as nmol/SAAPFPNA/min and IU/ml.

The enzymatic assays to assess the anti-tryptic potential and anti- chymotryptics of larvae were carried out adding 10 μl of bovine trypsin for the anti-tryptic and 10μl bovine chymotrypsin for the anti-chymotryptic, in order to determine whether they have inhibitory action on these enzymes. After addition of Tris-HCl 50 mM, pH 8.0, the respective substrates were added, continuing the incubation and reading at 410 nm, as described in the tryptic and chymotryptic assays (above paragraph). Three replicates were performed for each assay and sample. Reactions were read in Multiskan Go Microplate Reader at 410 nm.

### Statistical analysis

The results for each chemical analysis were individually analyzed. All analyzes were performed in triplicate and results were expressed as mean and standard deviation. Mean values between groups were compared by analysis of variance (ANOVA) and differences were compared by the Tukey test at significance level of p <0.05 using the software Statistica 8.0 [31], and Prism 3.0 [32].

## Results and Discussion

### Diet nutritional compositions

**Fig 1:**
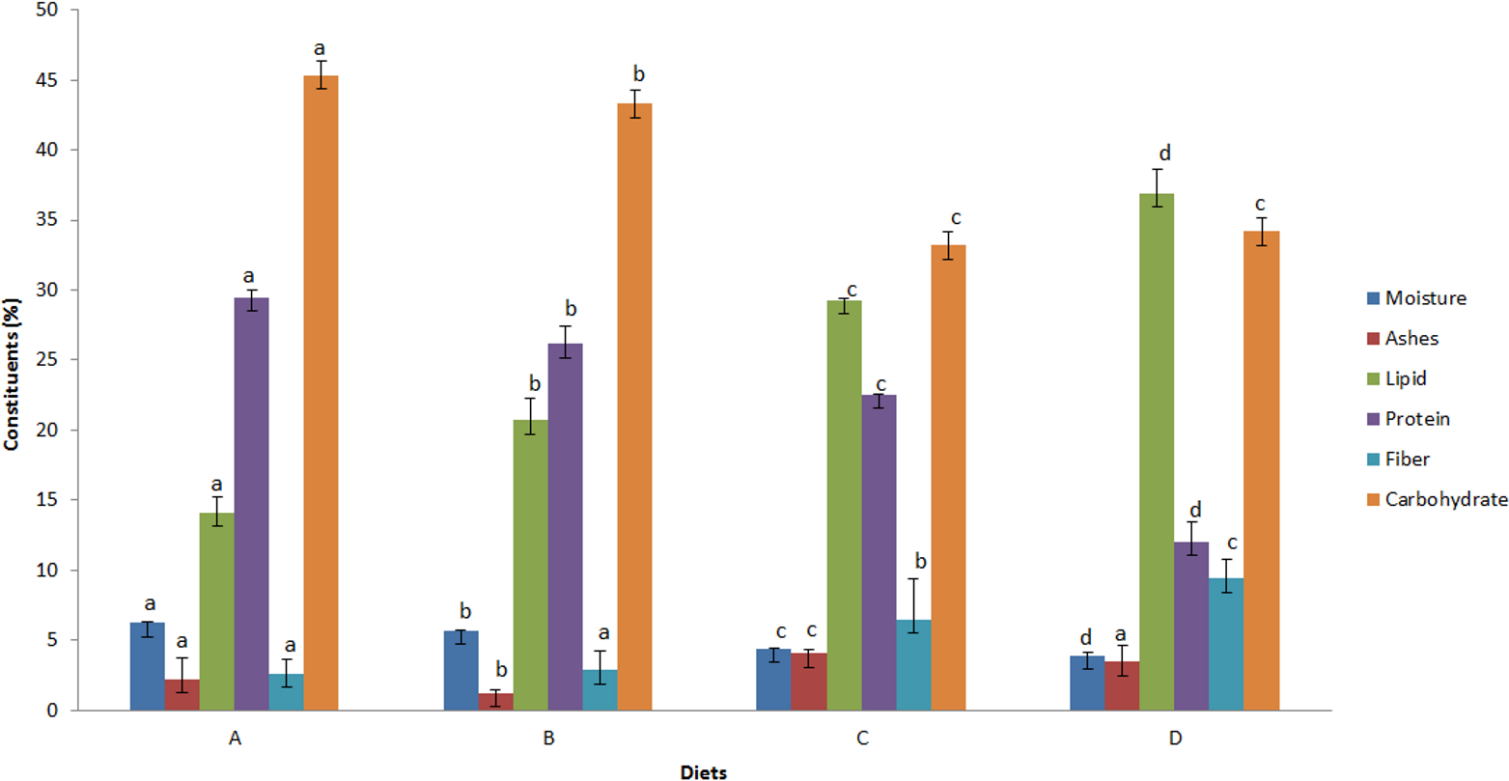
**Diet nutritional compositions (A, B, C and D) for growing *Tenebrio molitor* larvae (Coleopetera, Tenebrionidae).** (A) 50% wheat flour and 50% soybean flour (control diet); (B) 50% control diet and 50% bocaiuva pulp flour; (C) control diet 50% and 50% ground bocaiuva kernel; and (D) 50% ground bocaiuva pulp flour and 50% bocaiuva kernel. Different letters in the like columns for different diets differ significantly (p <0.05).

The diet nutritional compositions designed for growing mealworms is shown in Fig 1. The control diet (A) presented higher carbohydrates and protein content as expected, since it consisted of wheat and soybeans. The partial replacement of the control diet with bocaiuva flour and kernel (diets B and C) influenced the nutrient compositions, with significant (p <0.05) reduction in moisture, protein and carbohydrate levels and significant (p<0.05) increase in lipids and ashes amounts compared to the control diet A and diet B.

The diet D consisting of 50% bocaiuva pulp flour and 50% bocaiuva kernel (Fig 1) presented significant changes in lipid (36.93%) and protein (12.03%) content, and an increase in fiber content (9.43%). These changes were expected and are similar to previously reported bocaiuva pulp and almond nutritional components [23]. The total energy value in 100 g of the control diet (A) was 414 kcal and diets B, C and D were 450, 487, 517 kcal, respectively.

The insects survived throughout the larval stage on the four diets (A, B, C and D). However, after 130 days, reaching the adult stage, beetles from C and D diets died; this was probably due to the high lipid level (29.28% and 36.93%, respectively) (Fig 1). The high oil concentration favored the substrate agglomeration hindering aeration and movement of insects. The excess of oil can interfere in the beetle breathing, which is done through the trachea located in the abdomen of adult individuals [33].

The adult period (16 days) was not influenced by the diet consumed for insects of Diet A and B, agreeing with Aguilar-Miranda *et al*. [20], which states that the adult beetle life can vary from 4 to 17 days after copulation. Given these results, the nutritional composition were determined only for the larvae in diets A and B.

### Mealworm nutritional composition

Nutritional compositional of mealworms fed diet A and B are shown in Table 1. Mealworms from both diets had greater nutrient amounts compared to the conventional foods. The mealworms ashes content is higher than the chicken, beef, pork and fish [34]. The fixed mineral residue, inorganic chemicals needed in small amounts, contribute in formation of tissues, favoring the nerve impulse transmissions and muscle contraction, participating in the maintenance of acid-base balance, such as calcium, iron, magnesium, zinc and iodine [35].

**Table 1:** Nutritional composition of *Tenebrio molitor* (Coleopetera, Tenebrionidae) larvae grown on artificial diets A and B (photoperiod 10hLx14hD, T = 25°C) and conventional foods.

Samples	Moisture (g/100 g)	Ashes (g/100 g)	Lipid (g/100 g)	Protein (g/100 g)	Carbohydrate	
					Fibers (g/100 g)	Starch (g/100 g)
*T*. *molitor* Larvae (A)	51.91±0.78^a^	3.85±0.12^a^	39.05±2.06^a^	50.07±0.76^a^	18.84±1.01^a^	-
*T*. *molitor* Larvae (B)	52.78±0.87^a^	4.76±0.67^a^	40.45±0.42^a^	44.83±0.40^b^	13.42±1.38^b^	-
*T*. *molitor* Larvae **	56.27	3.54	50.15	40.98	-	-
Chicken*	63.6	2.2	57.42	42.58	N.A.	-
Egg*	75,6	4.24	44.88	48.51	N.A.	-
Beef *	52.7	1.9	67.23	35.31	N.A.	-
Pork*	36.9	2.22	48.02	47.58	N.A.	-
Fish *	59.9	2.49	48.88	49.63	N.A.	-

Values presented in dry matter with ± standard deviation, n = 3. (A) diet consisting of wheat flour and soybean flour, (B) diet consisting of 50% wheat flour and soybean flour and 50% bocaiuva pulp flour.

* TACO—Brazilian table of food composition, 2011. NA = not applicable.

** [36]. Means with different superscripts letters in the same column differ significantly (p <0.05).

The lipid percentage of the mealworms was 39.05% (Diet A) and 40.45% (Diet B). Lipids are important in the diet because they are vital to cell biological and structural functions and assist in fat-soluble vitamins transport, essential for body nutrition. They also enhance the food palatability by absorption and retention of flavors [37] and influence food texture given softness and crispness. Energetically, they are important because they produce 9 kcal/g when oxidized in the body. In some countries lipids represent 30–40% of total energy consumed in foods by humans [35, 37].

The digestibility of insect proteins is comparable with conventional meat [38, 39, 40]. The *T*. *molitor* larvae protein content (44.83 to 50.07%) was higher than foods known as rich in proteins such as chicken (42.58%) and beef (35.31%) (Table 1). However, there are reported values of *T*. *molitor* larvae total protein ranging from 49.8% to 76.14% [17]. Some authors [18] relate this variation in nutritional composition to the lack of standard methodologies for growing insects or for their feed.

The high protein concentration and digestibility are indicators that these insects can be used in food production for human consumption and in animal feed production. It was found that mealworms presents 44.09% of essential amino acids, which demonstrates the protein quality, thus they can be used as nutritional multi-mixtures [18].

Since the animal protein is superior to plant, the best protein supplements should include some animal protein [39]. Many of these products contain milk-derived protein; production of animals for their milk causes environmental impact much greater than the production of insects [39]. Products produced based on insects face a relatively low acceptability barrier, since they aim to attract consumers with nutritional and environmental awareness, and the protein source is not visible or its taste distinguishable (e.g. replacing the soybean powder by insect powder does not alter the product appearance, taste or texture) [39]. The insects used in the food industry can be a high quality protein ingredient for a high standard protein supplement.

Another important contribution of *T*. *molitor* larvae are fibers, mealworms in both diets had high fiber content (Table 1). Consumption of foods rich in fiber is related to cardiovascular risk reduction and reduced glucose and lipid levels related to decreased hyperinsulinemia. A high fiber consumption entails less risk of obesity development [41, 42].

This study demonstrates that the mealworms fed diet containing bocaiuva pulp flour (diet B) are sources of fiber, since the amount found was greater than 6.0 g/100 g, as established by the Ordinance No. 27/1998 of the National Health Surveillance Agency [43].

Considering the daily needs of an adult human related to minerals (2.8 g), protein (60 g), lipids (65 g) and fiber (30 g) [36], 30 g of larvae would attend, respectively, 51%, 23% 19% and 13% of these nutrients.

### Fatty acid composition and antioxidant activity

The fatty acid composition of the oil extracted from mealworms (diets A and B) is presented in Table 2. There were no significant differences between the fatty acids of the larvae on diets A and B, except for the presence (0.12%) of caprylic acid (C8: 0) in larvae on diet B.

**Table 2:** Fatty acid composition of the oil from *Tenebrio molitor* larvae (Coleopetera, Tenebrionidae) grown on artificial diets A and B (photoperiod 10hL x 14hE, T = 25°C).

Fatty acids	*T*. *molitor* larvae		Beef * (%)	Pork ** (%)
	Diet A (%)	Diet B (%)		
**Caprylic acid (C8: 0)**	-	0.12±0.01	0.50	-
**Lauric acid (C12: 0)**	0.53 ±0.01	0.64±0.01	0.30	0.01
**Myristic acid (C14: 0)**	4.26±0.09	4.35±0.04	4.0	0.16
**Palmitic acid (C16: 0)**	21.07±0.12	21.18±0.09	23.0	16.66
**Stearic Acid (C18: 0)**	6.88±0.13	6.95±0.11	-	3.16
**Arachidonic acid (C20: 0)**	0.46±0.01	0.48±0.01	-	0.55
**Palmitoleic acid (C16: 1)**	1.87±0.05	1.89±0.03	6.0	0.12
**Oleic acid (C18: 1)**	52.78±0.23	53.09±0.18	43.0	0.48
**Linoleic acid (C18: 2)**	11.45±0.29	10.78±0.21	2.0	-
**α- Linolenic acid (C18:3)**	0.18±0.01	0.23±0.01	2.0	1.12
**Eicosapentaenoic acid-CLA (C20:5)**	0.39±0.01	0.27±0.01		
**∑ SFA**	33.2	33.72	27.8	20.92
**∑ MUFA**	54.65	54.98	49.0	24.16
**∑ PUFA**	11.63	11.01	4.0	47.44

Values expressed in mean n = 3 and standard deviation. SFA: saturated fatty acids. MFA: monounsaturated fatty acids. PUFA: polyunsaturated fatty acids. (A) diet consisting of soybean flour and wheat flour, (B) diet consisting of 50% soybean flour and wheat flour and 50% *bocaiuva* pulp flour.

* [44].

** [45]. No significant differences were detected (p <0.05).

The mealworms (diets A and B) showed satisfactory levels of essential fatty acids, which are polyunsaturated and are not synthesized by the cells of mammals, and therefore have to be ingested in the diet. The essential fatty acids are linoleic (18: 2 n-6) and α-linolenic (18: 3 n-3) [37].

Despite mealworms (diets A and B) having less α-linolenic acid (0.18% and 0.23%) compared to beef (2.0%) and pork (1.12%), acid linoleic concentrations in these mealworms (11.45% and 10.78%) were higher than those conventional meats (2.0% and 0%).

These fatty acids act as precursors for the synthesis of long chain polyunsaturated fatty acids such as arachidonic acid, eicosapentaenoic and docosahexaenoic acid. These fatty acids are necessary to maintain, under normal conditions, cell membranes, brain function and the nerve impulse transmissions. They also participate in atmospheric oxygen transfer to the plasma, hemoglobin synthesis and cell division [46].

Polyunsaturated fatty acids from the omega-3 series (C18:3 and C20:5) and omega-6 (C18:2) may prevent of cardiovascular diseases and cancers [37]. High concentration of fatty acids in the oil affects its antioxidant activity, which is highly desired in the human diet [37].

The conjugated linoleic acid (CLA) is present in meat products and has shown antioxidant activity [44,45]. The CLA may decrease saturated fatty acids accumulation in cell membranes, making these membranes less susceptible to oxidation, with less potential to cause oxidative damage to cellular components [47,48].

Some other examples of antioxidants in meat include minerals, carnosine and glutathione [49]. The antioxidant activity of the oil from mealworms (diet A) measured by the ABTS method, which enables the use of hydrophilic and lipophilic extracts, was 4.5 μM Trolox/g, higher than conventional oils such as soybean (2.2 μM Trolox/g) and sunflower (1.17 μM Trolox/g) [35].

### Tryptic activity and anti-nutritional factors

The key process to protein digestion is mediated by proteolytic enzymes secreted by the pancreas, which directly assist digestion and activate other intestinal enzymes [50]. Among these enzymes, trypsin is found in digestive systems of many vertebrates that need to hydrolyze and absorb proteins.

The trypsin activity is fundamental in the hydrolysis process and consequently in proteins absorption, since these molecules are too large to be absorbed by the intestine [51]. The presence of trypsin inhibitors negatively influence the absorption and utilization of protein from the diet because the inhibitor binds to the trypsin preventing it from performing its function [51].

The trypsin activity of mealworm larvae (diet A) was 0.144±0.003 BAPNA nmol/min. The trypsin presence suggests that the consumption of such mealworms as a food can generate increased availability of amino acids. Corroborating this hypothesis, there was absence of anti-nutritional factors (anti- tryptic, and anti- chymotryptic) in mealworms.

## Conclusions

Results from this study indicate that artificial diets with added bocaiuva pulp flour could be a new source of biomass for rearing *Tenebrio molitor*. Artificial diets with fat content below 29% favored the development of *T*. *molitor* beetles. *T*. *molitor* larvae reared on artificial diet with added bocaiuva are good sources of protein and lipid, have significant concentrations of unsaturated fatty acids, antioxidant activity and absence of anti-nutritional factors. Larvae reared on diets with added bocaiuva pulp flour can be a highly nutritious food resource for food security and for use in protein supplements for athletes and people interested in increasing protein intake.
